# Intercrop-mediated inducibility affects direct defenses and plant resistance but not indirect defenses in maize

**DOI:** 10.3389/fpls.2026.1766071

**Published:** 2026-04-10

**Authors:** Juan Pablo Jordán, Anna DiPaola, Alison G. Power, Katja Poveda, André Kessler

**Affiliations:** 1 Department of Ecology and Evolutionary Biology, Cornell University, Ithaca, NY, United States; 2 Department of Entomology and Nematology, University of California, Davis, Davis, CA, United States; 3 Department of Entomology, Cornell University, Ithaca, NY, United States

**Keywords:** chemical defenses, companion cropping, induced responses, plant defense, plant secondary metabolites, intercropping

## Abstract

Functional intercropping aims to beneficially associate two or more plant species or varieties, simultaneously increasing plant diversity and the provisioning of ecosystem services. Although the benefits of diversified cropping systems are widespread and well documented, the underlying mechanisms of increased pest resistance and the relative contributions of different modes of plant defense remain unclear. Plant chemistry can mediate resistance to herbivores through toxic or antidigestive modes of action (direct defenses) or by providing host finding cues that recruit natural enemies that predate on herbivore populations (indirect defenses). Both direct and indirect defense can be elevated in response to previous herbivore damage leading to induced resistance. Here we address the question of how intercropping with four companion plants (alfalfa, bean, Desmodium, and red clover) affects the constitutive and induced expression of plant direct/indirect defenses and resulting herbivore resistance. We found that defensive plant secondary metabolite production of focal maize plants varies with both, previous herbivore damage (induction treatment) and the presence of an intercrop species. Intercropping - specifically with Desmodium - alters the expression of plant chemical defenses and increases plant resistance in 1) no-choice bioassays by reducing larval performance and 2) the incidence of damaged leaves at the field-scale experiment. Thereby some intercrop species do not only directly affect maize plant secondary metabolism but also alter how defensive metabolites are expressed in response to herbivory (intercrop-mediated induced responses). In contrast to direct resistance, the expression of indirect resistance did not vary with intercropping or herbivory suggesting that under realistic field conditions, direct defenses are more reliable as pest control mechanisms than chemical information-mediated indirect defenses. However, within-plant spatial separation of predation pressure suggests a role of vegetation structure in the efficiency of biocontrol. We present evidence that ‘intercrop-mediated induced responses’ is an integrated ecological mechanism determining the outcome of associational resistance (or susceptibility) and conclude that intercrop-mediated alterations of constitutive and herbivory-induced secondary metabolite production mediate increased associational resistance in diversified maize systems.

## Introduction

Functional intercropping is one of the most widely adopted crop diversification strategies (Beillouin et al., 2019), which intentionally and simultaneously grows two or more plant species in the same field (Brooker et al., 2015; Vandermeer, 1989). Spatially close heterospecific neighboring plants can, on one hand, increase the focal plant’s resistance to antagonists (i.e herbivores and pathogens), but in other cases may also heighten their susceptibility leading to the ecological phenomenon commonly known as associational effects (Barbosa et al., 2009). The resulting positive or negative plant-plant associational effects stemming from plant neighborhood - an inherent property of functional intercropping - will ultimately determine the successful provisioning of ecosystem services within the system. When plant neighborhood outcomes are optimized, intercropping provides a wide range of emergent properties including yield stability, improved soil health, and important to this study; increased herbivore resistance (Huss et al., 2022; Li et al., 2021). Various mechanisms have been proposed by which intercropping increases plant resistance to herbivory. For example, in the classic stimulo-deterrant strategy commonly known as “push-pull” (Khan et al., 1997; Pickett et al., 2014; Pyke et al., 1987), intercropping maize with select companion plants of the genus *Desmodium* provides associational resistance through the constitutive release of semio-chemicals that deter ovipositing adult moths while enhancing parasitoid attraction (Odermatt et al., 2024; Sobhy et al., 2022; but see Erdei et al., 2024). Recent evidence suggests additional intercrop-mediated mechanisms, such as the induction of direct resistance by the intercrop plant (Bass et al., 2024) and positive plant-soil feedbacks of growth and defense (Hu et al., 2025; Mutyambai et al., 2019). Furthermore, intercropping has long been associated with a general diversification of the interaction community and thus a general increase in abundance and diversity of natural enemies (Grof-Tisza et al., 2025), although derived pest control effects have rarely been reported (Poveda et al., 2008). In addition, these latest findings suggest intercrop-mediated alterations in the inducibility of direct and indirect defenses as major herbivore resistance-mediating mechanisms in companion crop systems, a hypothesis, we address with this study.

Defoliating and disease-vectoring arthropod herbivores are among the most impactful stressors for plants becasue they can negatively impact plant fitness in natural populations (Brown et al., 1987; Marquis, 1984; Mothershead and Marquis, 2000) and dramatically reduce yield in agricultural systems (Oerke, 2006; Savary et al., 2019). To mitigate such losses, modern agriculture predominantly relies on synthetic pesticides coupled with classic plant breeding and genetic transformation for increased constitutive resistance, which have proven environmentally unsustainable (Tilman et al., 2002). In addition to constitutively expressed herbivore resistance traits, plants have evolved the ability to trigger tailored responses to attacking herbivores by inducing heightened expression of defense-related morphological and physiological traits that increase the plants’ ability to fend off attackers and offset fitness costs (Agrawal, 1998; Karban and Baldwin, 1997). Both constitutive and inducible defensive traits can be broadly categorized into two main groups, differing in their mode of action: 1) direct defenses (Kessler and Baldwin, 2001) which increase the concentration of toxic, repellent, or detracting compounds across vulnerable tissue and 2) indirect defenses (Takabayashi and Dicke, 1996; Turlings et al., 1995), in which plants recruit natural enemies such as predators and parasitoids that exert top-down control on herbivore populations (Kessler and Heil, 2011).

Consequently, direct and indirect defenses mediate herbivore resistance in two basic ways, through the anti-nutritive or toxic effects provided by the chemical defense, or through the provision of chemical information (information-mediated indirect defenses) and/or food and shelter (resource-mediated indirect defenses) which facilitate host/prey search behavior and residence of natural enemies. Despite undergoing strong domestication and aggressive breeding practices, maize lineages retain broad chemical defenses such as benzoxazinoids (Gaillard et al., 2018; Maag et al., 2015), and there is mounting evidence of predator and parasitoids control of herbivore populations (Hoballah et al., 2002; Turlings et al., 1995). Furthermore, both direct and indirect defenses in maize are sensitive to previous herbivore damage causing induced responses which generally upregulate direct defenses and trigger volatile organic compound (VOC) release attracting predators and parasitoids. For this reason, throughout the manuscript we associate the term ‘induction’ with the prescribed herbivore damage treatment which measures the induced responses to said previous herbivore damage. Examples of plant-induced responses to herbivory are not constrained to maize but are widely documented across the plant kingdom (Heil et al., 2004; Thaler and Karban, 1997). However the biotic factors mediating the strength and direction of induction on both direct and indirect plant defenses and their cascading effects on herbivore resistance remain understudied.

This knowledge gap is especially apparent in high-input agricultural systems, which utilize pesticides (among other strategies) to avoid herbivore damage, in principle, excluding plant interactions with other organisms and so rendering induced resistance expression largely futile. However, the rapid evolution of pesticide resistance (Gould et al., 2018), the continuous range expansion of insect pests (Ma et al., 2021; Skendžić et al., 2021), and an increasing public awareness about the environmental and human health risks associated with synthetic pesticides (Goulson, 2013; Stanley et al., 2015) have intensified the search for efficient alternative pest control strategies, including an increased consideration of inducible resistance. The re-evaluation of inducible traits as tools in modern pest control derives from an increased understanding that plant induced responses expand the arena in which plant-organismal interactions are played out (Kessler, 2015) and the uncovering of additional induction-mediating factors such as neighboring plants (Holmes and Agrawal, 2021) and soil microbial communities (Cameron et al., 2013; Howard et al., 2020b). This additional induction-mediating factors allow plants to optimize those interactions to maximize fitness or yield outcomes. At the molecular level, induced resistance is regulated by a plant-behavioral integration of wound-induced endogenous, mostly phytohormonal, signals and by herbivore-derived chemical elicitors, which all interact in a signaling pathway crosstalk (Bostock, 2005). This combination of internal and external signaling allows for the specific fine-tuning of transcriptional and metabolic responses to a wide range of environmental elicitors that trigger comparable responses (Wu and Baldwin, 2010). Moreover, the plant signaling pathway crosstalk results in a transcriptional and metabolic reconfiguration of the plant that can be further altered by environmental conditions, such as other herbivores, neighboring plants, or interacting microbes (Schuman and Baldwin, 2016).

While direct (Lang et al., 2024; Mutyambai et al., 2019) and indirect defenses (Khan et al., 1997) are known to constitutively increase under intercropping practices, it remains unclear through what mechanisms and to what extent the inducibility of enhanced resistance is altered by the interaction of the focal crop plant with a functional intercrop and how inducibility contributes to intercrop-mediated signaling resulting in enhanced pest resistance. Moreover, plant-induced responses are highly context-dependent; for example, induction might not directly improve yield through herbivore defense but rather do so indirectly by triggering compensatory growth responses (Poveda et al., 2010). Further questions persist regarding the effectiveness of indirect defenses across spatial scales (Aartsma et al., 2017). For example, extraflorar nectary position (Mortensen et al., 2011) and mite domatia distribution across canopy architecture (Situngu and Barker, 2017) determine their defensive effect. However, the within-plant effectiveness of predation has not been previously evaluated. In other words, we have no indication if or how predation pressure differs across the plant, and if intercrop structural context or plant induced responses might change such spatial patterns. To address these knowledge gaps, we specifically ask the following question: How does intercrop identity and herbivory (induction) mediate the magnitude and direction of both direct and indirect plant defense? We assess how the effectiveness of indirect defenses (predator pressure) varies spatially within the plant and the reliability of direct and indirect resistance expression as pest control measures that can be manipulated with intercropping. We predict that intercropping affects both direct and indirect resistance to herbivory in a companion crop-specific manner and that intercropping affects the inducibility of resistance by herbivory and so alters the outcome of interactions between herbivore pests and the focal plant.

## Methods

### Field experimental design, wet biomass, and background herbivore damage

**Figure 1:**
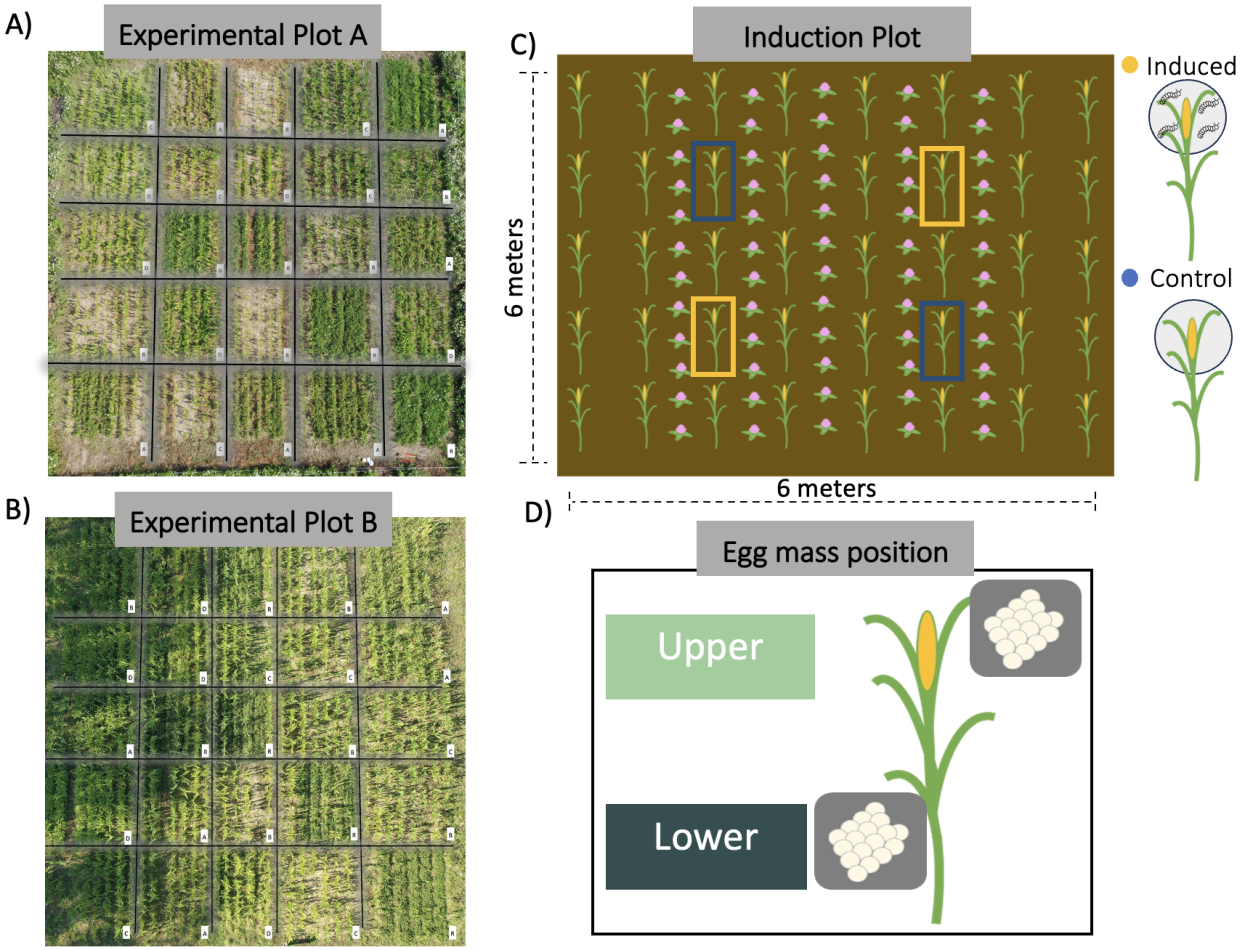
Overview of field experimental design and conceptual figures for induction treatment and egg mass location on focal maize plants. **(A, B)** show aerial photographs of experimental plots and their corresponding intercrop treatments. Letters on the bottom right corner specify the intercrop treatment of each subplot: Alfalfa (A), Beans (B), *Desmodium* (D), Red Clover (R), and maize monoculture control (C). Each experimental plot consisted of 25 subplots with 5 replicates per intercrop treatment for a total of 10 independent subplot replicates across both main experimental plots. The intercrop treatment was randomly assigned, and intercrops were manually planted in between the maize rows. **(C)** exemplifies the spatial layout of the induction treatment across sub-plots; yellow color indicates the induction treatment and blue color non-induced the control treatment. Plants were induced by placing three *Spodoptera frugiperda* larvae on the upper three fully expanded leaves, covered with a mesh bag, and left to damage the focal plants for three days. **(D)** depicts the within-plant distribution of egg masses on individual maize focal plants. The upper egg mass was placed in the second fully expanded maize leaf, while the lower egg mass was placed in the lowest non-wilted fully expanded leaf.

We established an intercropping field experiment at Dunlop meadow located in Brooktondale, New York, in which two 30 x 30 meter plots were converted to agricultural land-use following six years of natural succession (Howard et al., 2020a). Both plots underwent standard tillage (plowing/disking) and were further divided into 25 sub-plots of 6 x 6 meters each for a total of 50 subplots, creating 10 independent plot-level replicates per intercrop treatment. One subplot consisted of 7 rows of maize with four strips of intercrops planted within the maize rows resembling a strip intercropping design. The maize between-row spacing was 91 cm and the within-row spacing was 15 cm. Each subplot was randomly assigned an intercrop treatment or left as a maize monoculture with a horizontal two-row buffer between the sub-plots (**Figures 1A, B**). Maize rows were planted using a standard 4-row maize seeder, while the intercrops were planted using either a one-row inter-seeder or sown manually immediately after the maize was established in the field. The maize variety used as the focal plant was the Red Tail seed maize rt (49T61 Red Tail Seeds inc). Four different plant species were used as intercropping treatments: alfalfa (*Medicago sativa)*, red clover (*Trifolium pratense)*, common bean (*Phaseolus vulgaris)*, and showy tick-trefoil *(Desmodium canadense*). All intercrop seeds were obtained from Johnny’s Selected Seeds, except for *D. canadense*, which was acquired from Prairie Moon Nursery. Intercrop legumes were seeded between the five innermost rows of maize in each subplot at a rate of approximately 33.3 kg/ha within two days of maize planting. The field sites were naturally irrigated with available rainfall and weeded regularly by hand throughout the growing season with no additional fertilizer or external inputs utilized.

Nearing the end of the growing season (September 15^th^, 2023), we conducted a field survey to quantify the background level herbivory on maize plants across all experimental plots. We then quantified the proportion of damaged leaves by counting the total number of leaves and the number of damaged leaves for each maize plant of the two center-most rows of each intercrop subplot. A leaf was tallied as damaged if it visibly showed tissue defoliation in any part of that given leaf. Two weeks later (October 4^th^), we continuously harvested the center two maize rows and weighed the total wet biomass for a given row. That total plant mass measurement was then divided by the total number of plants in that given row representing the average wet weight per maize plant harvested as a proxy for yield.

### Induction experiment and quantification of indirect defenses

To measure how intercropping and herbivory-mediated induction interact to shape direct and indirect plant defenses in maize, we conducted an induction experiment across the previously established experimental plots. Within each subplot, four maize plants parallel to each other were chosen, of which two were subjected to a damage (induction) treatment and two were kept as controls (not induced; **Figure 1C**). Therefore, the experiment consisted of two phases: the induction treatment phase followed by the direct and indirect defense measurement phase. Plants in the induction treatment were induced by bagging three larvae (fourth-instar) of the generalist herbivore *Spodoptera frugiperda* on the three youngest fully-expanded leaves, while control plants were bagged, but no herbivore larvae were introduced leaving them undamaged. Larvae in the induction treatment were first introduced to the youngest fully expanded leaf, but the bag effectively bagged the top three leaves. All experimental maize plants were bagged with cloth mesh bags and closed at the bottom using cotton yarn. This bagging method limited herbivore movement and restricted damage to the three bagged leaves, while allowing normal respiration and gas exchange.

Each of the induction and control maize plant pairs were placed directly diagonal to each other and located within two intercrop rows to guarantee maize-intercrop interactions (See **Figure 1C**). After 96 hours of unrestricted feeding, each plant was unbagged, and leaf tissue samples collected *in-situ* for secondary metabolite analysis. Leaf samples were collected into a fast-prep vial, flash frozen using liquid nitrogen, and later stored in -80 C until chemical extraction (see section of indirect defenses below for details). The remaining three bagged leaves were collected into individual bags with a moist paper towel and taken back to the laboratory for use in resistance bioassays. We then deployed sentinel egg masses to measure predation pressure across our experimental plants. To do so, we deployed two sentinel egg masses per plant; the lower egg mass was paperclipped to the lowest non-wilted maize leaves (~30cm from the soil surface), and the upper egg mass was paperclipped to the highest fully-expanded maize leaf with a visible collar (~ 1.7 meters above soil surface) (**Figure 1D**). There was a 24-hour shift in the induction and measurement phase across the two experimental plots A and B since it was not possible to conduct all required fieldwork in a single day. However, we applied the same protocol with identical relative timing of induction, larvae removal, and egg mass deployment for the plot B, but shifted 24 hours later, beginning egg mass deployment. Sentinel egg masses were photographed before and after field deployment, which allowed us to quantify the number of predated eggs by counting the outgoing number of eggs and the remaining number of eggs using the photo processing software image J. Freshly oviposited egg masses of *S. frugiperda* (Fall Army Worm) were obtained from Benzon Research (Pennsylvania, USA) for both the sentinel egg masses and for rearing the inducing herbivores for the induction phase.

To passively monitor the community of natural enemies, we installed yellow sticky card traps at the center of subplots on wooden stakes at 1.5 m above the soil surface for four days. We sampled two subplots per intercrop species within each replicated field, beginning on 26 July 2023 in the experimental field A and on 31 July in the experimental field B. Sticky cards were collected at the end of the experiment and taken back to the laboratory for natural enemy identification. We also actively sampled the natural enemy community in the plots by using aspirators to manually collect natural enemies on one row of maize and one row of intercrops within every subplot. Experimental field A was sampled on July 30th and the experimental field B on August 4th 2023 (See **Supplementary Tables 12**, **13**).

### Secondary metabolite analysis and quantification of direct defenses

Leaf tissue samples were collected from the third fully expanded leaf (outside the mesh bag) of each experimental maize plant using a sterilized razor, flash frozen in liquid nitrogen, and stored at -80 °C awaiting chemical extraction. Samples were chemically extracted by adding 1 ml of methanol (Sigma-Aldrich, St. Louis, MO, USA) and ~ 0.9 g of grinding beads (BioSpec R, Zirconia/Silica 2.3 mm) into a FastPrep tube containing standardized ~150–200 mg of leaf tissue. Tissue samples were then disrupted and homogenized by two cycles of 60 s at 6 m/s using the FastPrep (MP Biomedicals R, Solon, Ohio, USA) tissue homogenizer. Between each breakdown cycle, samples were sonicated for 5 minutes at room temperature (~24 °C). Homogenized tissues were then centrifuged twice at 4°C for 15 min at 14, 000 rpm (Eppendorf centrifuge with 30 tubes, fixed angle rotor: 20, 800 × g) whereby 700 μL of the supernatant was removed after the first cycle and 500 μL in the second cycle, and transferred into clean Eppendorf tubes carefully avoiding any impurities. Finally, 100 μL of the remaining supernatant was transferred into individual high-performance liquid chromatography (HPLC) vials. To analyze the secondary metabolite profile of each focal maize plant, 15 μL of the supernatant was injected into an Agilent 1100 HPLC coupled to a Diode Array Detector with a Gemini C18 reverse phase column (3 μm, 150 × 4.6 mm, Phenomenex, Torrance, CA, USA). The elution system consisted of aqueous 0.25% phosphoric acid (H3PO4) and methanol (CH_3_OH), which were pumped through the column at a rate of 0.7 ml/min with increasing concentrations of methanol: 0–5 min, 0–20% methanol; 5–35 min, 20–95% methanol. Resulting chromatograms were aligned using the chromatographR package (Bass, 2023) and the resulting peak table was manually inspected for potential column splitting and normalized by sample weight. To further identify the specific secondary metabolites that explain variation upon induction, we used commercially available internal standards from Sigma-Aldrich (USA) to identify previously known defense-related compounds, specifically Benzoxazinoids 2, 4-dihydroxy-7-methoxy-1, 4-benzoxazin-3-one (DIMBOA), 6-methoxy-2-benzoxazolinone (MBOA).

### Plant herbivore resistance bioassay

To evaluate maize resistance to herbivory, we utilized no-choice feeding assays using larvae of the Fall Army Worm (*Spodopetra frugiperda*). Approximately 30 eggs were separated into individual rearing cups with cabbage looper diet, allowing hatched larvae to feed unrestrictedly for a period of 5 days. Following the feeding period, third instar larvae were transferred into individual 5 cm diameter plastic petri dishes with non-nutritive agar for a starvation period of 9 hours. We then recorded the initial weight of each larva and offered a 2 cm diameter leaf disc previously collected from the third fully expanded leaf of maize focal plants originating from different field intercrop and induction treatments. The bioassay and secondary metabolite sample corresponded to the same leaf origin. The larvae were allowed to feed on the leaf discs for 96 hours and were kept under room temperature (24 °C) with a 16:8-hour photoperiod. After the feeding period concluded, we recorded the final weight and the area of leaf disk consumed using the leaf byte app (Getman-Pickering et al., 2020). We had 20 bioassay replicates per treatment for a total sample size of 180 individual bioassays.

### Statistical analysis

#### Agricultural performance metrics and resistance bioassay

To test for the effect of intercrop treatment on average plant wet weight (yield) and the proportion of damaged leaves, we fitted a linear mixed model (LMM) and a generalized linear mixed model (GLMM), respectively. The LMM assumed a Gaussian distribution, while the GLMM leveraged a binomial distribution with a logit-link to model leaf damage probabilities, which were then converted to proportions for ease of interpretation. Model structure included ‘intercrop’ treatment as a fixed effect and a nested plot/sub-plot random effect. All data analysis was done in R version 4.5.1 (R Core Team, 2024) and plots visualized using the ggplot2 package (Wickham, 2016). All mixed models were fitted as implemented in the glmmTMB package (McGillycuddy et al., 2025). Estimated marginal means were modelled and pairwise *post hoc* tests were conducted using the emmeans package (Lenth, 2024). We evaluated the significance of individual treatments and their interactions using a type three sums of squares test (ANOVA); plots are annotated with the resulting p-values.

We assessed herbivore resistance using three metrics derived from the no-choice larval feeding bioassay: 1) the percent of leaf area consumed, 2) the weight gained within the trial duration, and 3) larval biomass accumulation efficiency (i.e. weight gained per area of leaf eaten). All data was analyzed by fitting GLMM’s using the following model structure: ‘intercrop’ and ‘induction’ as fixed effects with their interaction, followed by ‘plot/subplot’ as nested random effects. The model used to analyze the percent of leaf area consumed used a ‘beta-regression’ approach for continuous proportions (Douma and Weedon, 2019). The other two models for weight gained and larval biomass assumed a normal (Gaussian) distribution.

#### Overall plant secondary metabolite profiles and direct defenses

We utilized a permutational analysis of variance (PERMANOVA) to test for overall changes in maize secondary metabolite profiles as a function of intercrop and induction treatments using the *adonis2* function in the Vegan package with plot coded as ‘strata’ (Anderson, 2001; Oksanen et al., 2024). To visualize these differences, we used non-metric multidimensional scaling NMDS ordination (metaMDS; Vegan), plotting individual replicates and mean points surrounded by standard deviation ellipses, which visualizes changes across chemical space. To further understand how intercropping and induction interact to shape the expression of chemical defenses and therefore predict plant resistance to herbivory, we fitted linear regression models to the peak area of individual plant secondary metabolites previously identified as predictor of the three main resistance measures from the non-choice bioassay. These models had the same structure with fixed and interaction effects of intercrop and induction, with the added peak area as a predictor. Models were plotted using the *predict_response* function as implemented in the ggeffects package (Lüdecke, 2018).

#### Indirect defenses

To analyze predation pressure as a measure of indirect defenses, we fitted a GLMM, which followed the same basic model structure as described above for resistance bioassay analysis, with the addition of ‘position’ as a fixed effect with interaction to test for the spatial distribution effect (above or below) in predation pressure. Overdispersion was tested using the *check_overdispersion* function of the performance package (Lüdecke et al., 2021). To account for overdispersion in our model, we added an observation-level random effect (Harrison, 2014).

## Results

### Intercrop effect on yield and background level herbivore damage

**Figure 2:**
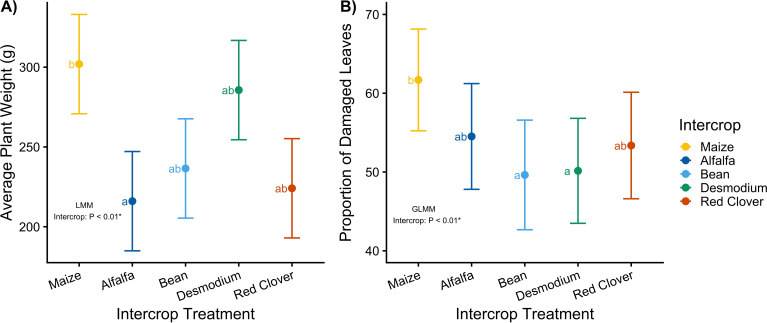
Mean (± SEM) of plant wet mass as a proxy for yield **(A)** and mean (± SEM) proportion of damaged leaves **(B)** of field intercropped maize. Letters indicate statistically significant differences between means in a pairwise comparison using the Tukey method after fitting a linear mixed model **(A)** and a logistic regression model **(B)**. Plots are annotated with p-values from a type III partial sums of squares ANOVA.

Intercrop treatment (i.e. companion plant species) significantly affects maize yield (*LMM, X^2^ = 13.39, p < 0.01*; **Figure 2A**) and field-level herbivore damage (*GLMM_logistic_, X^2^ = 14.82, p = 0.005*; **Figure 2B**). Intercropping with alfalfa decreased maize biomass by 28.5% compared to the maize monoculture control (**Figure 2A**; **Supplementary Table 1**) while bean, *Desmodium*, and red clover intercrop treatments did not affect yield. Interestingly, *Desmodium* intercropping showed similar performance to maize controls with a minimal yield reduction of only 5.4%. The proportion of damaged leaves was significantly reduced under bean and *Desmodium* intercropping (**Figure 2B**). Irrespective of the intercrop identity, plot diversification through intercropping resulted in a 16% decrease in the proportion of damaged leaves (**Supplementary Table 2**). Herbivore damage is widespread and ubiquitous across maize plants in our experiment, with mean values clustering at fifty percent indicating that at least half of the scored leaves sustained some level of defoliation (**Figure 2B**).

### Effects of intercrop and herbivore damage (induction) on maize secondary metabolite profiles

**Figure 3:**
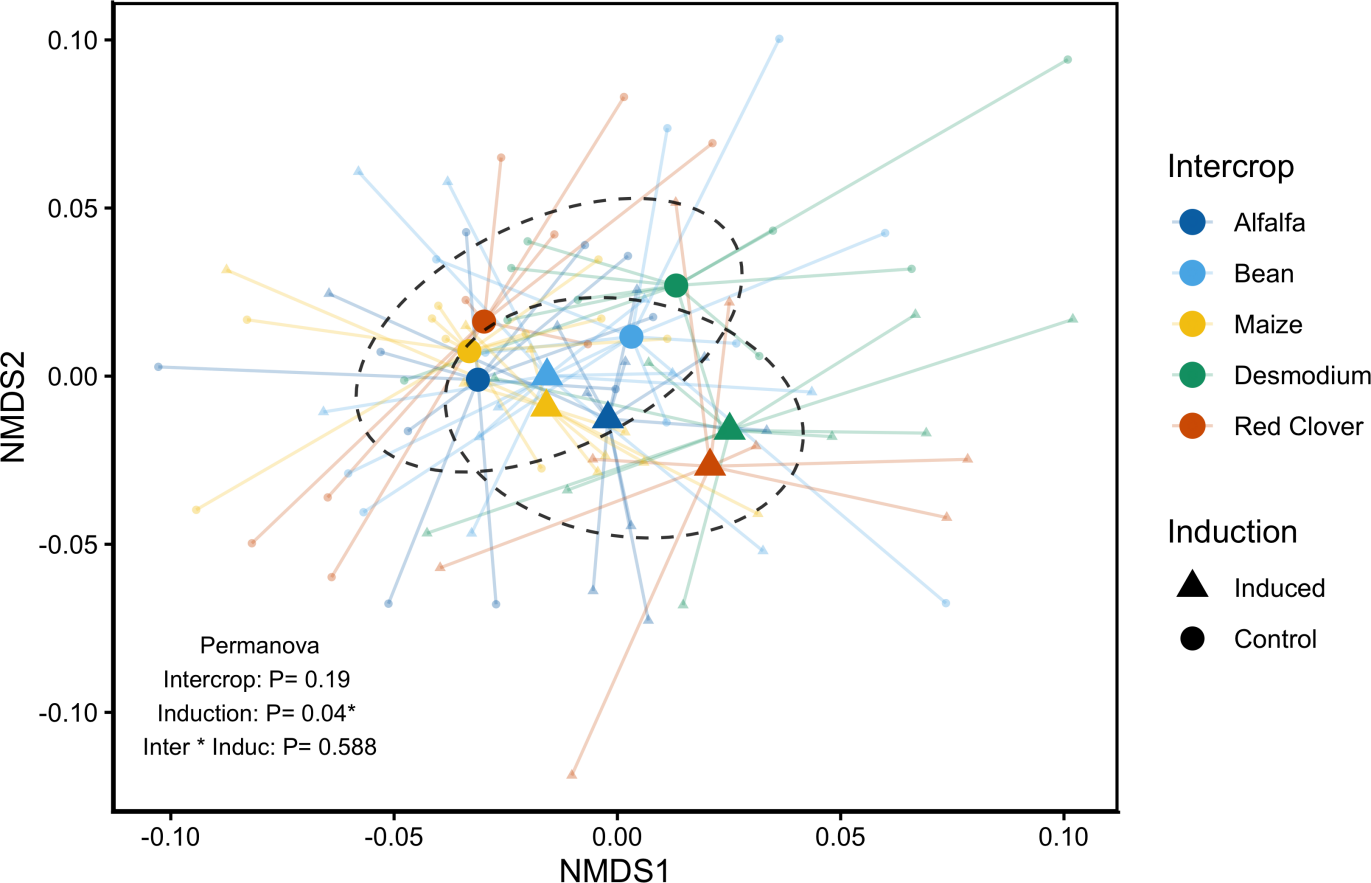
Non-metric multidimensional scaling (NMDS) of focal maize plant secondary metabolite profiles in response to intercropping (Alfalfa, Bean, Maize, Red Clover, and *Desmodium*) and induction treatments. Centroids correspond to the mean distance across each independent vector represented in the chemical space (stress value = 0.145), and dotted lines represent one standard deviation of the mean. Non-induced control plants are denoted with circular-shaped centroids, while induced plants are distinguished by a triangle-shaped centroid. Plots are annotated with p-values from a permutational analysis of variance test (PERMANOVA). Standard deviation ellipses represent the distribution of each group in chemical space.

Permutational analysis of variance (PERMANOVA) revealed that the induction treatment alone (*F_1, 95_ =* 2.302*, p = 0.045*) significantly changes the overall foliar maize secondary metabolite profile, distinctly clustering across dimensional space (**Figure 3**). The intercrop identity did not have a measurable effect (*F_1, 95_ =* 1.23*, p = 0.*191) nor was there a significant interaction between these factors (*F_1, 95_ = 0.869, p = 0.*588; **Supplementary Table 3**).

### Effects of intercrop identity and herbivore damage (induction) on maize resistance to herbivory

**Figure 4:**
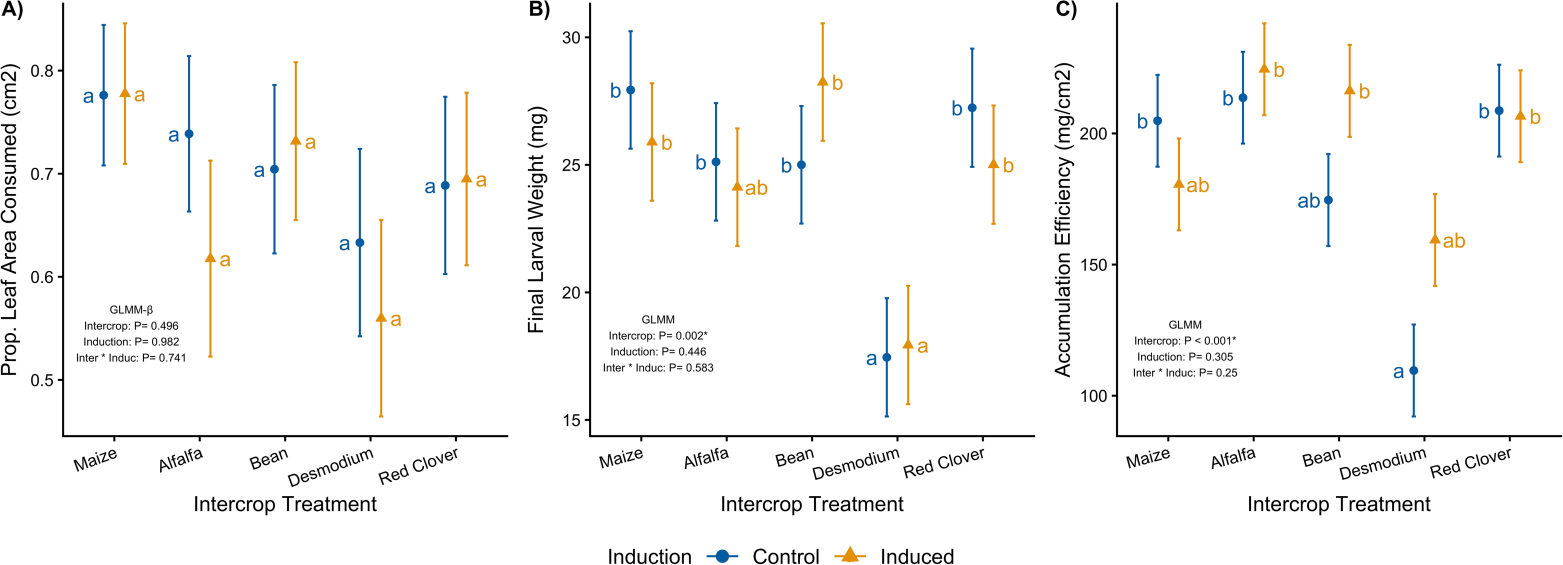
Maize resistance to *Spodoptera frugiperda* larvae measured by the mean (± SEM) proportion of leaf area consumed **(A)**, mean (± SEM) final larval weight **(B)**, and mean (± SEM) body mass accumulation efficiency **(C)**. Control treatments are indicated by the blue circle and induction treatment by the yellow triangle denoting the mean and standard error values for each treatment group. Letters indicate statistically significant differences between means in a pairwise comparison using a Tukey *post hoc* test after fitting generalized linear mixed models. Plots are annotated with p-values from a type III partial sums of squares ANOVA testing for the individual and interactive effects of intercrop and induction treatments.

There was no measurable effect of intercrop or induction on the proportion of leaf area consumed (*GLMM_Inter_, X^2^ = 3.379, p = 0.496*; GLMM_Induc_, X^2^ = 0.12, p = 0.982; GLMM_Inter*Induc_, X^2^ = 1.968, p = 0.741;**Figure 4A**; **Supplementary Tables 4**, **5**). Final larval weight, however, was strongly affected by intercrop identity (*GLMM_Inter_, X^2^ = 147.113, p = 0.002*), with larvae reared on *Desmodium* being significantly smaller than those reared on maize, alfalfa, or bean (**Figure 4B**). Neither induction (*GLMM_Induc_, X^2^ = 17.463, p = 0.446*) nor its interaction (GLMM_Inter*Induc_, X^2^ = 2.849, p = 0.583) with intercrop influenced final larval weight (**Supplementary Tables 6**, **7**). Body mass accumulation efficiency (mg/cm² leaf consumed) also differed among intercrops (*GLMM_Inter_, X^2^ = 136.932, p < 0.001*), with the lowest observed in larvae feeding on non-induced *Desmodium*-intercropped plants (**Figure 4C**). Again, induction by herbivory had no significant effect nor was there an interaction between both factors (GLMM_Induc_, X^2^ = 24.445, p = 0.305; GLMM_Inter*Induc_, X^2^ = 7.576, p = 0.25; **Supplementary Tables 8**, **9**). Although the effect of intercrop and herbivory-mediated induction did not show a significant interaction, it is important to note that herbivore damage increased the caterpillars’ body mass accumulation efficiency when intercropping with alfalfa, bean, and *Desmodium.* Therefore, the resulting accumulation efficiency is context-dependent and while the effects are not statistically significant, they can be biologically relevant. Taken together, our results show that intercropping treatment significantly influenced herbivore performance across two metrics of plant resistance, while induction herbivore damage had no measurable effects across all three herbivore performance metrics (**Figures 4A–C**).

### Intercrop-mediated induction of maize benzoxazinoids (direct defenses) and their effect on herbivore resistance

**Figure 5:**
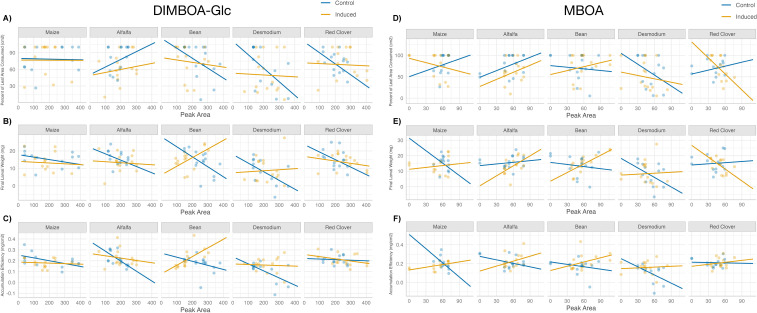
Three metrics of plant resistance {final larval weight [**(A)**+**(D)**], percent of leaf area consumed [**(B)**+**(E)**], and accumulation efficiency [**(C)**+**(F)**]} as a function of DIMBO-Glc and MBOA tissue concentrations [**(A–C)** DIMBOA and **(D–F)** MBOA] for each intercrop treatment and control. Herbivory treatment (induction) linear predictions and data points are in yellow, while values of undamaged control plants are denoted in blue.

The magnitude and direction of induced resistance across three metrics of herbivore performance (percent of leaf area consumed, final larval weight, and accumulation efficiency) were correlated with the production of two known defensive benzoxazinoid compounds DIMBOA-Glc (**Figures 5A–C**) and MBOA (**Figures 5D–F**) and further mediated by the interaction between intercrop species and induction. There was a significant interaction between peak area and *Desmodium* intercrop (β = –0.119 ± 0.051, *t* = –2.31, *p* = 0.022), suggesting that only under *Desmodium* intercropping *S. frugiperda* larvae decreased the percent of leaf area consumed (**Figure 5A**). The final larval mass was affected by a three-way interaction between DIMBOA-Glc, bean intercrop, and induction (β = 0.049 ± 0.018, *t* = –2.681, *p* = 0.0084), indicating that the strength and direction of induced resistance is contingent not only on the intercrop identity, but induced responses to herbivory can inversely change the outcome of resistance. In other words, under bean intercropping induction results in associational susceptibility increasing larval mass, while non-induced control plants under bean intercropping provide associational resistance, decreasing larval growth (**Figure 5B**). Nevertheless, we found an overall negative relationship of increased DIMBOA-Glc and the mass accumulation efficiency of *S. frugiperda* larvae (β = -0.00013 ± 0.000064, *t* = –2.041, *p* = 0.0437; **Figure 5C**). Similar to the final larval weight, accumulation efficiency was also significantly affected by a three-way interaction between DIMBOA-Glc, bean intercrop, and induction (β = 0.00047 ± 0.00023, *t* = –2.024, *p* = 0.0477; **Figure 5C**). Our results show that relationships between benzoxazinoid abundance and larval performance varied by intercrop species and were often contingent on induction status.

MBOA leaf tissue concentration, intercrop species, or herbivore damage had no effect on the percent of leaf area consumed by *S. frugiperda* larvae (**Figure 5D**). However, final larval mass varied in a three-way interaction between MBOA concentration, induction treatment, and red clover as intercrop treatment (β = -0.588 ± 0.258, *t* = –2.278, *p* = 0.0245; **Figure 5E**). There was a significant overall effect of increased MBOA peak area (β = -0.005 ± 0.0024, *t* = –2.029, *p* = 0.0447) and induction (β = -0.375 ± 0.155, *t* = –2.415, *p* = 0.0172) in decreasing the accumulation efficiency of *S. frugiperda* larvae (**Figure 5F**). Furthermore, there was a significant two-way interaction between MBOA peak area and induction, where induction increased overall larvae accumulation efficiency (β = 0.0059 ± 0.0025, *t* = 2.346, *p* = 0.02). Similar patterns of context dependence were observed for the accumulation efficiency, with a marginally significant three-way interaction between MBOA peak area, induction, and red clover intercrop (β = 0.332 ± 0.178, *t* = 1.859, *p* = 0.065). By comparing across three metrics of larval performance, we uncover finer-scale differences of induced responses and their cascading effects on maize resistance and how these are structured by intercrop species and previous herbivory.

### Effect of intercrop, herbivore damage (induction), and sentinel egg mass position on predation pressure (indirect defenses)

**Figure 6:**
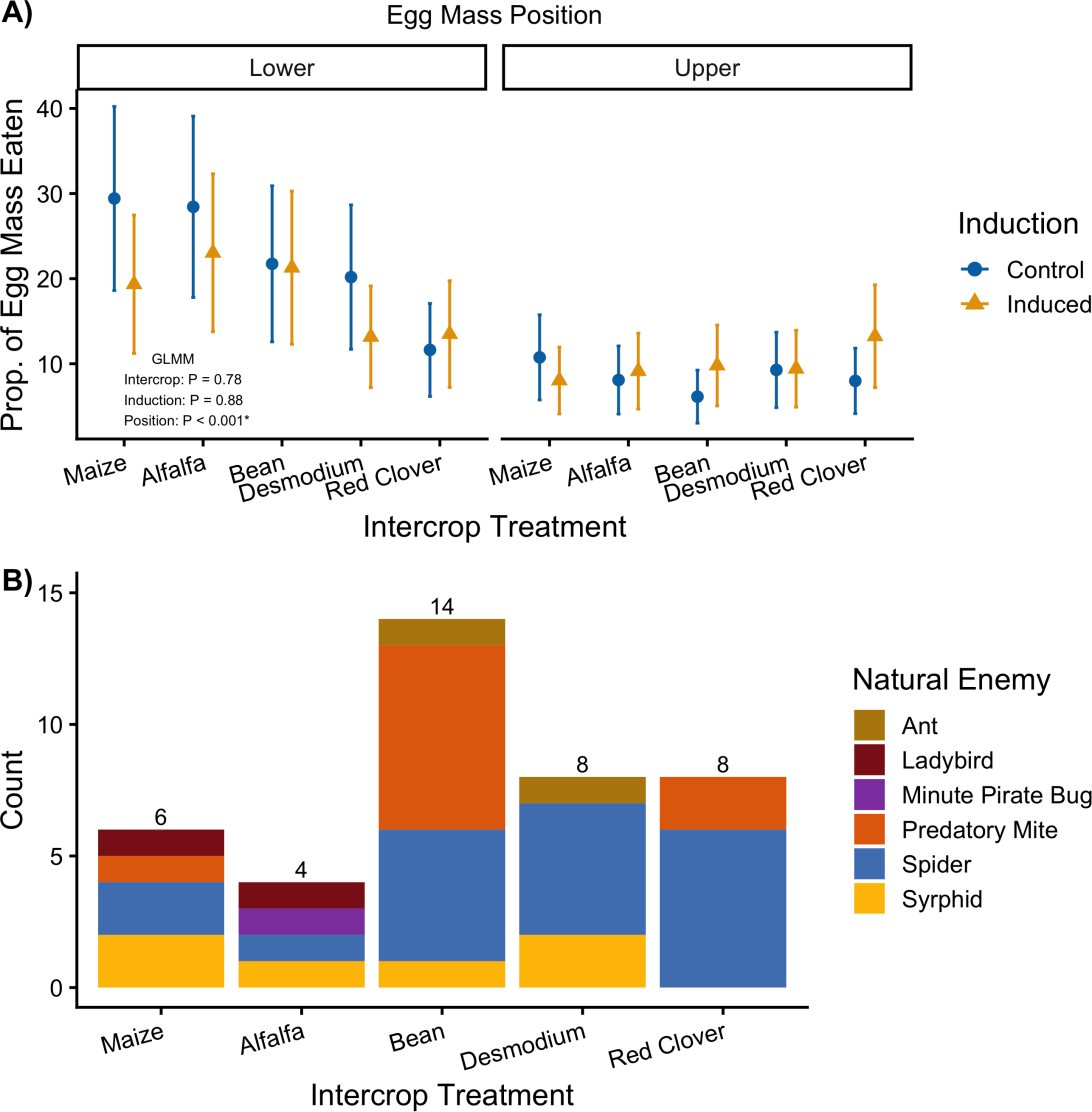
**(A)** Mean (± SEM) proportion of sentinel egg mass eaten as a measure of predation pressure on maize plants exposed to differnt intercrops and herbivory (induced) in the lower an upper plant of the focal maize plant. Control treatments are indicated by the blue circle and herbivory treatment by the yellow triangle. Plots are annotated with p-values from a type III partial sums of squares ANOVA testing for the individual effects of intercrop, induction, and spatial position treatments. **(B)** Stacked bar plot showing the total abundance and composition of the natural enemy community across intercropping treatments.

Egg mass predation was not affected by intercrop (*GLMM_Inter_, X^2^ =, p = 0.78*) or induction treatment (*GLMM_Induc_, X^2^ =, p = 0.88*). Interestingly, there was a significant spatial separation of egg predation *(GLMM_Position_, X^2^ =, p < 0.001;***Figure 6A**; **Supplementary Tables 10**, **11**), in which sentinel egg mass placed in the lowest fully expanded leaf suffered from higher predation compared to sentinel egg masses placed in the top fully expanded leaf of the focal maize plants. Passive monitoring of the natural enemy community showed tha bean intercropping had the highests abundance of natural enemies, in which spiders and predatory mites were the most prevalent. Alfalfa showed the lowest abundance of natural enemies, closely followed by the maize control (**Figure 6B**).

## Discussion

Induced responses to environmental factors such as herbivory or neighbors allow plants to adaptively adjust their phenotypes to varying and challenging conditions (Mertens et al., 2021). We found that both the expression of defensive secondary metabolites and herbivore resistance vary with intercrop species and is crucially mediated by herbivore damage (induction). This result suggests that external cues coming from neighboring plants as well as endogenous cues in response to herbivore damage, shape induced responses. In contrast, the expression of indirect resistance varied between low and high positions within the maize plant but not with intercrop species or induction, suggesting a stronger dependence of indirect defenses on external environmental factors rather than the integration of cues at the metabolic level. These results touch on two major ecological concepts with implication for the application of chemical ecology principles in agriculture: 1) the integration of environmental cues during the regulation of plant metabolic and pest resistance responses and, 2) the interaction between specific plant responses to environmental stressors and the ecological context. We propose the use of “intercrop-mediated induced responses” when describing physical or physiological changes in response to both induction and intercropping, and “intercrop-mediated induced resistance” when referring to the resulting effects on plant resistance. This nomenclature expands the traditional definition of induced responses and plant neighborhood effects, allowing the use of precise language to describe this particular phenomenon widely applicable across diversified cropping systems.

### Potential integration of neighboring plant cues mediating intercrop-induced resistance

Emerging research has shown that plants have the ability to integrate and respond to multiple sources of environmental information including: chemical cues such as volatile organic compounds (VOCs) or soil-borne root exudates emitted by neighboring plants, spectral cue variation such as alterations in red:far-red light ratios which are indicative of plant neighborhood, and plant-soil feedback-mediating assemblages of soil microbial and fungal communities (Anten and Chen, 2021; Chautá and Kessler, 2022; Escobar-Bravo et al., 2024). The intercrop-mediated induced resistance we have observed in our study can theoretically be mediated by all of the latter mechanism, and when considering the variation in benzoxazinoid expression across maize tissues and intercrop treatments, the intercrop-specific differential integration of environmental information is particularly obvious. However, theoretical and empirical work suggest that upon herbivore damage induced responses trigger measurable, often standardized responses (Kaplan et al., 2008). These measurable and standardized responses include but are not limited to the upregulation of defensive compounds, increased emission of natural-enemy attracting VOCs (Tamiru et al., 2011), and primed gene expression (Kessler et al., 2006). Our results show significant variation in benzoxazinoid DIMBOA and MBOA concentrations as a function of both intercrop and herbivore damage. Thus, the resulting secondary metbolism and associated resistance effects can be interpreted as a response continuum with intercrop-mediated and herbivory-mediated signaling as tunable dials that affect the eventual maize plant defense phenotype. In other words, if benzoxazinoid expression is sensitive to induction and intercropping alike, the difference in benzoxazinoid concentrations could be explained by the strength to which the focal maize plant was able to pick up or tune-into the intercrop’s cues. Another possibility is that the variation in benzoxazinoid concentrations correlates with a third factor facilitated by the intercrop. Specifically, companion plant-triggered and soil microbial community-mediated plant-soil feedbacks may further affect the defense phenotype. For example, arbuscular mycorrhizal fungi colonization (AMF) of maize roots has shown to mediate inducibility of plant resistance (Kempel et al., 2010) and the upregulation of defensive metabolites (Jung et al., 2012; Tao et al., 2016; Vannette and Hunter, 2013). Therefore, it could be predicted that the benzoxazinoid expression patterns could be explained by differences in AMF colonization of maize roots that varies by the intercrop identity. Plant chemistry, in turn, affects soil microbial community structure.

A recent study in maize expanded on previous findings showing that maize-emmitted green leaf volatiles induced changes in the secondary metabolism of neighboring plants, which in turn, triggered positive plant-soil feedbacks that enhance resistance in subsequently grown maize plants through changes in the soil microbiome (Guo et al., 2025; Hu et al., 2025). Thereby, green leaf volatiles that are rather ubiquitously released in response to tissue damage by most plant species (Ameye et al., 2018) relay relatively unspecific information making it a generalizable mechanism of plant-neighborhood inducing resistance to herbivory. Information about the neighboring plant identity and what herbivore species is attacking are likely encoded in the further specific bioactive VOCs or in the overall composition of a neighboring plant’s VOC bouquet. Such metabolic phenotype-altering effects of complex VOC bouquets can explain plant resistance responses to heterospecific, undamaged neighbors as has been observed in response the *D. canadense* and bean neighbors in this study. Indeed, aboveground exposure of maize plants to the headspace VOCs of *D. uncinatum* affect defensive metabolism and herbivore resistance. However, related studies also suggested a significant role of below-ground chemical signaling as well as an alteration of responses that resulted from the exposure the spectral information provided from light reflected off of leaves on neighboring plants (Bass et al., 2024). On one hand, simple priming of the soil with *D. uncinatum* resulted in a plant-soil feedback-induced change in maize secondary metabolism and resistance (Mutyambai et al., 2019, 2024). On the other hand, plants are well known to alter their defense metabolite production when exposed to shifts in the red: far red light ratio emitted off of neighboring plants (Chautá and Kessler, 2022; Izaguirre et al., 2006). Therefore, a model in which the focal maize plant integrates both (above and below-ground) chemical and spectral cues emitted from the neighboring intercrop is most likely to explain the high intercrop specific alteration in overall maize secondary metabolism and herbivore-induced resistance we found in this study. This is further supported by the fact that we do not see a generalized effect of intercropping *per se*, and that intercropping and cover cropping practices result in fundamentally different maize secondary metabolite profiles (Jordán and kessler, 2026), which has far-reaching implications for the adoption of intercrop practices in agriculture.

Examples of integration of chemical and spectral cues are currently restricted to studies of interactions between conspecific plant neighbors (Chautá and Kessler, 2022; Escobar-Bravo et al., 2022), intensifying the call for a more thorough investigation of the secondary metabolism-altering effects of heterospecific neighbors and the underlying signaling mechanisms. The high intercrop species specificity observed in this study provides a promising starting point for such a mechanistic analysis of heterospecific plant-plant communication. The ecological effects have previously been reported from only one system: the interaction between wild sagebrush (*Artemisia tridendata*) and coyote tobacco (*Nicotiana attenuata*) (Karban et al., 2000). While this latter example found VOC-mediated information transfer to be sufficient to explain the resistance-inducing effect of sagebrush VOCs on coyote tobacco (Kessler et al., 2006), it also found strong competitive interactions that could explain part of the metabolic shifts (Karban et al., 2003) and so emphasize the likely increased importance of the environmental context for the ecological consequences of plant induced responses.

### Plant induced responses and the environmental context

The environmental context can affect the ecological consequences of plant induced responses in two basic ways. First, a changing context can further alter the way phenotypic changes are induced. Second, an altered environmental context may change how plant induced metabolic changes affect the outcome of plant interactions with other organisms. The first is a result of the nature of indcuibility in which every additional information about the environment can alter the plant’s endogenous signaling pathway crosstalk (Kessler and Baldwin, 2002). For example, competition imposed by an intercrop could compromise the potential positive effects of intecrop-mediated resistance and is thus a crucial factor to be considered when evaluating the viability of intercropping as a pest control strategy (Cuny et al., 2025). Intercrop species may affect growth and secondary metabolism in the focal maize plant by changing the availability of resources. Nutrient availability (Mason et al., 2022) and water stress (Ngumbi and Ugarte, 2021) have both been shown to mediate plant defensive secondary metabolism and resistance to herbivory, but specific effects are not consistent with high variability dependent on the magnitude of stress (Kamps and Poelman, 2024). Therefore, the success of diversified cropping systems largely hinges on first reducing plant competition through agronomic management, followed by the strategic choice of functional intercrop informed by the plant’s ecology to synergize interactions through complementary or functional plant traits (Brooker et al., 2015). For this reason, legumes are a popular choice as functional intercrops used in various diversified cropping systems given their nitrogen-fixing capabilities which dampens nutrient stress potentially decreasing competition (Bybee-Finley and Ryan, 2018). However, our results suggest that this assumption is not necessarily true. While beans, red clover and *D. canadense* intercrops more or less maintained the productivity of non-intercropped maize monocultures, intercropping with alfalfa resulted in reduced maize biomass. It is also remarkable that none of the intercrops used in our experiment increased yield relative to maize monocultures suggesting intercrop-mediated resistance gains cannot outweigh the increased competition.

Little is known on the co-varying defensive traits (the chemical environment) in response to intercropping or induction following herbivore damage. Plants possess multiple chemical defenses which increase redundancy potentially acting as additional layers of defense if one might be overcome by an attacking herbivore. In maize, possible co-varying defensive traits include a diversity of benzoxazinoid-derived compounds (Robert and Mateo, 2022), physical defenses such as trichomes (Acevedo et al., 2021), and other anti-digestive complexes such as protease inhibitors (Tamayo et al., 2000). However, redundant defense traits are predicted to be strongly selected against if plant defenses are costly. Seminal papers indicate that inducing plants incur important fitness and ecological costs (Baldwin, 1998; Heil and Baldwin, 2002) and therefore defense strategies like direct and indirect defenses are likely to trade off. Furthermore, there is evidence that information-mediated indirect defenses (i.e. mediated through VOC compounds) are less reliable than resource-mediated indirect defenses (e.g. extrafloral nectaries) (Kessler and Heil, 2011). This apparent trade-off seems unlikely in maize given that both direct and indirect defenses remain strongly expressed (Tamiru et al., 2011; Zhou et al., 2018). However, emerging evidence suggests that plants might have strategic layered programing in which ‘cheaper’ defensive traits are deployed first followed by more ‘costly’ ones in a scale depended manner with different induction thresholds as cost saving strategies to herbivore attack (Bass, 2025; Wan et al., 2025). However, given that plants grown in agricultural systems have gone through extensive domestication and their selection agents shifted from natural to artificial selection partially negatively impacting defensive traits (Whitehead et al., 2017), the known ecological patterns of induced responses might not be applicable. Beyond the speculative nature of generalizable patterns of induced responses in applied systems and the correlative focus of our analysis, an integrative evaluation of co-varying defensive traits to induction and intercropping will provide insight into multiple traits shaping resistance in diversified cropping systems.

### Reliability and within-plant spatial structuring of indirect defenses

Biocontrol agents can similarly be viewed as a context-dependent resource when considering the effects of traits that mediate indirect defenses. Maize was one of the first plant species for which VOC-mediated indirect resistance was reported. Parasitoid wasps were found to be attracted by herbivory-induced VOCs and had significant impacts on herbivore mortality (Turlings et al., 1995). However, the application of indirect defense mechanisms depends on two context-providing factors; first, the presence of predators in sufficient numbers and second, the predators response to plant alarm signals, which have to be sufficiently reliable to attract predators in the background of environmental noise (Kessler, 2015). The actual availability of sufficient numbers of predators with a significant impact on herbivore populations has proven to be one of the crucial obstacles to a field application on indirect defenses. While an application in closed systems, such as greenhouses or biological control augmentation in the field have found commercial use (van Lenteren et al., 2018), reliability on indirect defenses can now been viewed as limited by the nature of the relative indirect interaction between a biocontrol agent and the plant (Kessler and Heil, 2011). Our data here can be interpreted within this framework. While both constitutive and inducible direct resistance is expressed in a intercrop-specific manner, the expression of indirect resistance did not vary with intercrop or induction treatments. Interestingly, the fact that predation levels varied with the position on the plant and with the experimental populations is in support of the hypothesis that the expression of indirect resistance strongly depends on the consistent availability of a biocontrol agent, which apparently was not given in our experiment. Interestingly, studies that tried to locally increase the density of biocontrol agents often succeeded with that increase in predator or parasitoid numbers but rarely demonstrated an effect on herbivory and almost never on yield (Poveda et al., 2008), suggesting additional obstacles to a indirect resistance application.

One of those obstacles is the reliability of the VOC cues in information-mediated indirect resistance (Kessler and Heil, 2011). This results from the fact that a plant’s constitutive and induced (defense) phenotype provide information to interacting organisms (Kessler, 2015) and different environmental contexts can inflict different types of noise to that information (Kessler and Kalske, 2018). Thereby, the information provided by direct resistance traits, with a herbivore making a relatively unfiltered food choice based on the repellency and toxicity of the plant tissue, is inherently less prone to environment-inflicted noise than the VOC cues providing the information to prey-searching predators and parasitoids. This unreliability is best illustrated with the fact that intercropping with *D. uncinatum* results in a plant-soil feedback-mediated induction of VOC emissions in maize that resemble those on herbivore-damaged plants. While these *de facto* constitutive emissions of an alarm cue can repel herbivory, they become, without a herbivore prey being present, a type of misinformation to foraging predators because of the lack of an association of the cue with a prey.

Intercrop species differ in their functional traits inherently varying the provisioning of predator-attracting resources like nectar, pollen, shelter, or alternative. Any potential resources an intercrop could provide a predator may be less valuable depending on their proximity to similar resources outside the experimental plot (Pierre et al., 2023). Furthermore, dependent on the location of the field, certain locations could offer more moisture, shade, and favorable temperatures relative to its immediate surroundings or the replicate field creating a spatial gradient of potential attraction. We expected intercrops to provision resources and influence the constitutive indirect defenses of maize plants at the subplot scale, but we did not see any effects of either treatment. Within subplots, predators’ host-finding could be hampered by olfactory cues from an intercrop plant interfering with their detection of these induced maize volatiles. Shimoda et al., 2012 showed that the presence of floral volatiles as a background olfactory cue reduced the attractiveness of herbivore-induced volatile emissions to predatory mites. Alternately, some intercrops varied in their phenological stage raging from vegetative state, flowering, and fruiting during the induction experiment, potentially diluting or concentrating resources and therefore confounding the response of predators in the field (Bugg et al., 1987). Both mechanisms are possible, since the volatile organic compound background and directed mobility of natural enemies is known to influence predator behavior at these scales (Glassmire et al., 2024).

Interestingly, we found that predation pressure is spatially structured egg masses lower on the plant received significantly more predation than those in the upper part. This result highlights the importance of pest oviposition location on predation rates. Intercrops could be hypothesized to augment predator densities by added structural refugia closer to the ground since maize significantly outgrows the intercrops. We expected that induction would erase a hypothetical spatial distribution of predation pressure by providing predators with information of suitable prey prompting them to increase mobility within the plant structure. However, there could be conflicting selection on this behavior since, ground-dwelling predators could experience higher parasitation through increased exposure higher up the plant. This remains an open line of research and the high variability in our experiment highlights that farmers and researchers must parse out the relationship between an intercrop species and natural enemy predation across different spatial contexts to support robust conservation biocontrol.

## Conclusion

Induced responses to neighboring plants and herbivory mediate differences in the secondary metabolism of focal maize plants (**Figure 3**). The intercrop companion plant identity impacts herbivore resistance. Specifically, *Desmodium* intercropping increases herbivore resistance by decreasing herbivore final mass and mass accumulation efficiency (**Figure 4**). Our results suggest that induced responses to previous herbivore damage interacts with the intercrop companion plant to uniquely structure effects on plant resistance through changes in expression of defensive secondary metabolites (**Figure 5**). Furthermore, predation pressure measured as a proxy for indirect defenses was structured by the within-spatial positioning (low or high) of the egg masses on the focal plant and was not affected by herbivory or intercropping treatment (**Figure 6**). We interpret this lack of clear indirect resistance effects of intercropping and herbivory as resulting from highly variable influences of the environmental context and the lack of reliability of the associated plant-natural enemy signaling on the outcome of plant-mediated predator prey interactions (Heil, 2008). The relative differences between direct and indirect resistance effects also suggest that and have previously been understood as a limited reliability of information-mediated indirect resistance traits as viable pest control options in a plant’s natural defense arsenal (Kessler and Heil, 2011). Too many moving parts make specifically chemical information-mediated indirect resistance traits less predictable relative to direct defense traits. This does not mean that indirect resistance traits can’t function well in pest control. It means that functionality is much more dependent on the plant varieties, the cultivation practices, the environmental conditions and the availability of predators and thus are far more knowledge-based and dependent on more manipulative, knowledge-dependent intervention. In contrast, our results suggest that direct pest resistance can be manipulated with specific intercropping application and can be enhanced and fine-tuned by using crop varieties that can induce strong resistance in response to herbivory.

Maize holds immense agronomic and economic value while arguably being the leading plant model organism for understanding the basic ecological interactions and the underlying molecular mechanisms of direct and indirect defenses (Erb et al., 2015; Sorg et al., 2025). Knowledge on the mechanisms increasing pest control of maize diversified cropping systems are therefore central to the long-term success of maize production through ecological intensification. All the potential mechanisms through which intercropping provides increased plant resistance (and other ecosystem services) are not mutually exclusive, most likely arising from a combination of interacting factors within the agroecosystems.

## Data Availability

Data supporting the findings of this study are openly available through the “Cornell eCommons” repository under the manuscript’s title. R scripts are available through a GitHub repository: https://github.com/jpj73/Intercrop-Induction.
