# Supplementary material for: Intercrop-mediated inducibility affects direct defenses and plant resistance but not indirect defenses in maize
Source: Front Plant Sci. 2026 Apr 10;17:1766071. doi: 10.3389/fpls.2026.1766071 (PMC13106325; doi:10.3389/fpls.2026.1766071)
Supplement: Supplementary file 1 [file Table1.docx]

***Supplementary Material***

***For manuscript: “Intercrop-mediated Inducibility Affects Direct Defenses and Plant Resistance but not Indirect Defenses in Maize”***

Supplementary Table 1. Model summary statistics for a linear mixed model predicting the average wet weight as a proxy for biomass.

| **Treatment** | **Estimate** | **SE** | **95% CI** | **p-value** |
| --- | --- | --- | --- | --- |
| **Intercrop** |  |  |  |  |
| *Maize* | — | — | — |  |
| *Alfalfa* | -85.9 | 29.8 | -146, -25.9 | **0.006** |
| *Bean* | -65.4 | 29.8 | -125, -5.39 | **0.033** |
| *Desmodium* | -16.3 | 29.8 | -76.3, 43.7 | 0.587 |
| *Red Clover* | -77.8 | 29.8 | -138, -17.8 | **0.012** |
| Abbreviations: CI = Confidence Interval, SE = Standard Error | | | | |

Supplementary Table 2. Model summary statistics for a logistic regression predicting the proportion of damaged leaves.

| **Treatment** | **Estimate** | **SE** | **95% CI** | **p-value** |
| --- | --- | --- | --- | --- |
| **Intercrop** |  |  |  |  |
| *Maize* | — | — | — |  |
| *Alfalfa* | -0.295 | 0.141 | -0.572, -0.019 | **0.036** |
| *Bean* | -0.491 | 0.154 | -0.793, -0.189 | **0.001** |
| *Desmodium* | -0.470 | 0.135 | -0.736, -0.205 | **<0.001** |
| *Red Clover* | -0.341 | 0.146 | -0.627, -0.056 | **0.019** |
| Abbreviations: CI = Confidence Interval, OR = Odds Ratio, SE = Standard Error | | | | |

Supplementary Table 3. Permutational analysis of variance (PERMANOVA) of maize leaf tissue overall plant secondary metabolite profiles with intercrop and induction treatments as fixed predictors and their interaction term.

| *Variables* | *Df* | *Sum Of Sqs* | *R2* | *F* | *P-value* |
| --- | --- | --- | --- | --- | --- |
| Intercrop | 4 | 0.121 | 0.051 | 1.234 | 0.190 |
| Induction | 1 | 0.057 | 0.024 | 2.302 | **0.039** |
| Inter*Induc | 4 | 0.085 | 0.036 | 0.869 | 0.588 |
| Residual | 86 | 2.111 | 0.889 | - | - |
| Total | 95 | 2.374 | 1.000 | - | - |

Supplementary Table 4. ANOVA type III (partial sums of squares) table for the proportion of leaf area consumed.

| *Treatment* | *Chisq* | *Df* | *P-value* |
| --- | --- | --- | --- |
| Intercrop | 3.379 | 4 | 0.497 |
| Induction | 0.12 | 1 | 0.983 |
| Inter * Induc | 1.968 | 4 | 0.742 |

Supplementary Table 5. Model summary statistics for a generalised linear mixed model (beta-regression) predicting the proportion of leaf area consumed.

| **Treatment** | **Estimate** | **SE** | **95% CI** | **p-value** |
| --- | --- | --- | --- | --- |
| Intercrop |  |  |  |  |
| Maize | — | — | — |  |
| Alfalfa | -0.204 | 0.398 | -0.985, 0.577 | 0.609 |
| Bean | -0.375 | 0.402 | -1.16, 0.412 | 0.350 |
| Desmodium | -0.697 | 0.403 | -1.49, 0.092 | 0.083 |
| Red Clover | -0.449 | 0.412 | -1.26, 0.358 | 0.275 |
| Induction |  |  |  |  |
| treatmentInduced | 0.009 | 0.402 | -0.780, 0.797 | 0.983 |
| Intercrop * Induction |  |  |  |  |
| Alfalfa * treatmentInduced | -0.568 | 0.577 | -1.70, 0.562 | 0.324 |
| Bean * treatmentInduced | 0.126 | 0.567 | -0.986, 1.24 | 0.825 |
| Desmodium * treatmentInduced | -0.314 | 0.568 | -1.43, 0.799 | 0.580 |
| Red Clover * treatmentInduced | 0.021 | 0.579 | -1.11, 1.16 | 0.972 |
| Abbreviations: CI = Confidence Interval, SE = Standard Error | | | | |

Supplementary Table 6. ANOVA type III (partial sums of squares) table for final larval weight.

| *Treatment* | *Chisq* | *Df* | *P-value* |
| --- | --- | --- | --- |
| Intercrop | 147.113 | 4 | **0.002** |
| Induction | 17.463 | 1 | 0.446 |
| Inter * Induc | 2.849 | 4 | 0.583 |

Supplementary Table 7. Model summary statistics for a generalised linear mixed model predicting the final larval weight.

| **Treatment** | **Estimate** | **SE** | **95% CI** | **p-value** |
| --- | --- | --- | --- | --- |
| Intercrop |  |  |  |  |
| Maize | — | — | — |  |
| Alfalfa | -0.003 | 0.003 | -0.008, 0.003 | 0.313 |
| Bean | -0.003 | 0.003 | -0.008, 0.003 | 0.293 |
| Desmodium | -0.010 | 0.003 | -0.016, -0.005 | **<0.001** |
| Red Clover | -0.001 | 0.003 | -0.006, 0.005 | 0.804 |
| Induction |  |  |  |  |
| treatmentInduced | -0.002 | 0.003 | -0.007, 0.003 | 0.446 |
| Intercrop * induction |  |  |  |  |
| Alfalfa * treatmentInduced | 0.001 | 0.004 | -0.006, 0.008 | 0.782 |
| Bean * treatmentInduced | 0.005 | 0.004 | -0.002, 0.013 | 0.163 |
| Desmodium * treatmentInduced | 0.003 | 0.004 | -0.005, 0.010 | 0.505 |
| Red Clover * treatmentInduced | 0.000 | 0.004 | -0.008, 0.007 | 0.960 |
| Abbreviations: CI = Confidence Interval, SE = Standard Error | | | | |

Supplementary Table 8. ANOVA type III (partial sums of squares) table for accumulation efficiency.

| *Treatment* | *Chisq* | *Df* | *P-value* |
| --- | --- | --- | --- |
| Intercrop | 136.932 | 4 | **< 0.001** |
| Induction | 24.445 | 1 | 0.277 |
| Inter * Induc | 7.576 | 4 | 0.108 |

Supplementary Table 9. Model summary statistics for a generalised linear mixed model predicting the accumulation efficiency of Spodoptera frugiperda larvae.

| **Treatment** | **Estimate** | **SE** | **95% CI** | **p-value** |
| --- | --- | --- | --- | --- |
| Intercrop |  |  |  |  |
| Maize | — | — | — |  |
| Alfalfa | 0.009 | 0.025 | -0.040, 0.057 | 0.722 |
| Bean | -0.030 | 0.025 | -0.079, 0.018 | 0.222 |
| Desmodium | -0.095 | 0.025 | -0.144, -0.047 | **<0.001** |
| Red Clover | 0.004 | 0.025 | -0.045, 0.052 | 0.875 |
| Induction |  |  |  |  |
| treatmentInduced | -0.024 | 0.022 | -0.068, 0.019 | 0.277 |
| Intercrop * Induction |  |  |  |  |
| Alfalfa * treatmentInduced | 0.035 | 0.032 | -0.027, 0.097 | 0.266 |
| Bean * treatmentInduced | 0.066 | 0.032 | 0.004, 0.128 | **0.037** |
| Desmodium * treatmentInduced | 0.074 | 0.032 | 0.012, 0.136 | **0.019** |
| Red Clover * treatmentInduced | 0.022 | 0.032 | -0.040, 0.084 | 0.483 |
| Abbreviations: CI = Confidence Interval, SE = Standard Error | | | | |

Supplementary Table 10. ANOVA type III table for proportion of sentinel egg mass eaten (predation) as a measure of predation pressure.

| *Treatment* | *Chisq* | *Df* | *P-value* |
| --- | --- | --- | --- |
| Intercrop | 1.767 | 4 | 0.779 |
| Induction | 0.023 | 1 | 0.879 |
| Position | 19.703 | 1 | **< 0.0001** |
| Interc*Induc | 2.218 | 4 | 0.696 |
| Inter*Posit | 4.043 | 4 | 0.400 |
| Induc*Posit | 1.094 | 1 | 0.296 |
| Inter*Induc*Posit | 0.077 | 4 | 0.999 |

Supplementary Table 11.  Model summary statistics for a generalised linear mixed model predicting the proportion of sentinel egg mass eaten as a measure of predation.

| ***Treatment*** | ***Estimate*** | ***SE*** | ***95% CI*** | ***p-value*** |
| --- | --- | --- | --- | --- |
| *Intercrop* |  |  |  |  |
| *Maize* | *—* | *—* | *—* |  |
| *Alfalfa* | *-0.048* | *0.624* | *-1.27, 1.18* | *0.939* |
| *Bean* | *-0.406* | *0.637* | *-1.65, 0.842* | *0.524* |
| *Desmodium* | *-0.500* | *0.626* | *-1.73, 0.728* | *0.425* |
| *Red Clover* | *-1.15* | *0.630* | *-2.39, 0.083* | *0.067* |
| *Induction* |  |  |  |  |
| *Control* | *—* | *—* | *—* |  |
| *Induced* | *-0.553* | *0.622* | *-1.77, 0.666* | *0.374* |
| *Position* |  |  |  |  |
| *Lower* | *—* | *—* | *—* |  |
| *Upper* | *-1.24* | *0.622* | *-2.46, -0.023* | ***0.046*** |
| *Intercrop * Induction* |  |  |  |  |
| *Alfalfa * Induced* | *0.270* | *0.882* | *-1.46, 2.00* | *0.760* |
| *Bean * Induced* | *0.526* | *0.900* | *-1.24, 2.29* | *0.559* |
| *Desmodium * Induced* | *0.041* | *0.884* | *-1.69, 1.77* | *0.963* |
| *Red Clover * Induced* | *0.723* | *0.895* | *-1.03, 2.48* | *0.419* |
| *Intercrop * Position* |  |  |  |  |
| *Alfalfa * Upper* | *-0.266* | *0.891* | *-2.01, 1.48* | *0.765* |
| *Bean * Upper* | *-0.206* | *0.901* | *-1.97, 1.56* | *0.820* |
| *Desmodium * Upper* | *0.336* | *0.885* | *-1.40, 2.07* | *0.704* |
| *Red Clover * Upper* | *0.826* | *0.886* | *-0.912, 2.56* | *0.352* |
| *Induction * Position* |  |  |  |  |
| *Induced * Upper* | *0.231* | *0.885* | *-1.50, 1.97* | *0.794* |
| *Intercrop * Induction * Position* |  |  |  |  |
| *Alfalfa * Induced * Upper* | *0.185* | *1.26* | *-2.29, 2.66* | *0.884* |
| *Bean * Induced * Upper* | *0.303* | *1.28* | *-2.20, 2.81* | *0.812* |
| *Desmodium * Induced * Upper* | *0.297* | *1.26* | *-2.16, 2.76* | *0.813* |
| *Red Clover * Induced * Upper* | *0.162* | *1.26* | *-2.31, 2.64* | *0.898* |
| *Abbreviations: CI = Confidence Interval, SE = Standard Error* | | | | |

Supplementary Table 12. Natural enemy abundance data from manual aspirators of experimental plot A.

| Natural enemy transects: Experimental plot A | |
| --- | --- |
| Intercrop species | Arthropod taxa |
| Alfalfa | *Coleomegilla maculata* (8 indiv.) |
|  | *Coccinella septempunctata* (1 indiv.) |
|  | *Hippodamia glacialis* (1 indiv.) |
|  | *Orius insidiosus* (1 indiv.) |
|  | Parasitoid wasps (1 Superfamily Chalcidoidea, 1 unknown) |
| Bean | *Coleomegilla maculata* (2 indiv.) |
|  | *Orius insidiosus* (2 indiv.) |
|  | Spider (1 Agelenidae) |
|  | Parasitoid wasps (1 Phytigidae, 2 Braconidae, 1 Diapriidae) |
| Red clover | *Propylea quatuordecimpunctata* (1 indiv.) |
|  | *Orius insidiosus* (1 indiv.) |
|  | Parasitoid wasps (1 Ichneumonidae, 1 Phytigidae, 1 Superfamily Chalcidoidea) |
| *Desmodium canadense* | Opiliones (1 indiv.) |
|  | Parasitoid wasps (1 Superfamily Chalcidoidea) |
| Maize monoculture | *Orius insidiosus* (1 indiv.) |
|  | Spider (1 Salticidae) |

Supplementary Table 13. Natural enemy abundance data from manual aspirators of experimental plot B.

| Natural enemy transects: Experimental plot B | |
| --- | --- |
| Intercrop species | Arthropod taxa |
| Alfalfa | *Coleomegilla maculata* (5 indiv.) |
|  | *Adalia decempunctata*(1 indiv.) |
|  | *Propylea quatuordecimpunctata* (2 indiv.) |
|  | *Orius insidiosus* (2 indiv.) |
|  | Predatory hemiptera (1 Geocoridae, 1 Nabidae) |
|  | *Spider (Theridiidae)* |
|  | *Ants (2 indiv.)* |
|  | Parasitoid wasps (1 Superfamily Chalcidoidea, 1 Platygastridae) |
| Bean | *Coleomegilla maculata* (3 indiv.) |
|  | *Propylea quatuordecimpunctata* (1 indiv.) |
|  | Ants (2 indiv.) |
|  | Spiders (3 Thomasidae, 2 Salticidae) |
|  | Parasitoid wasp (1 unknown family, damaged) |
| Red clover | *Coleomegilla maculata* (3 indiv.) |
|  | *Propylea quatuordecimpunctata* (1 indiv.) |
|  | *Orius insidiosus* (1 indiv.) |
|  | Parasitoid wasps (1 Platygastridae) |
|  | Spider (1 Araneidae) |
| *Desmodium canadense* | *Hippodamia parenthesis* (1 indiv.) |
|  | Parasitoid wasps (1 Superfamily Chalcidoidea) |
|  | Predatory hemiptera (1 Nabidae) |
|  | Ants (3 indiv.) |
|  | Opiliones (1 indiv.) |
|  | Spiders (2 Thomisidae, 1 Araneidae) |
| Maize monoculture | *Coleomegilla maculata* (1 indiv.) |
|  | *Propylea quatuordecimpunctata* (2 indiv.) |
|  | Earwig (*Forficula auricularia*) |
|  | *Orius insidiosus* (1 indiv.) |
|  | Spiders (1 Salticidae, 1 Theridiidae) |
|  | Ants (2 indiv.) |
|  | Parasitoid wasps (2 Superfamily Chalcidoidea, 1 Braconidae) |
